# Effect of Mechanical Stretch on the DNCB-induced Proinflammatory Cytokine Secretion in Human Keratinocytes

**DOI:** 10.1038/s41598-019-41480-y

**Published:** 2019-03-26

**Authors:** Seunghee Oh, Hyewon Chung, Sooho Chang, Su-Hyon Lee, Seung Hyeok Seok, Hyungsuk Lee

**Affiliations:** 1 School of Mechanical Engineering, Yonsei University, Seoul, 03722 South Korea; 2 Department of Microbiology and Immunology, and Institute of Endemic Disease, College of Medicine, Seoul National University, Seoul, 03080 South Korea; 3 Global Technology Center, Samsung Electronics, Co., Ltd., Suwon, 16677 South Korea; 4 R&D Institute, Biosolution Co., Ltd, Seoul, 01811 South Korea

## Abstract

Skin is exposed to various physico-chemical cues. Keratinocytes, a major component of the skin epidermis, directly interact with the surrounding extracellular matrix, and thus, biochemical and biophysical stimulations from the matrix regulate the function of keratinocytes. Although it was reported that inflammatory responses of skin were altered by an applied mechanical force, understanding how the keratinocytes sense the mechanical stimuli and regulate a cytokine secretion remains unclear. Here, we designed a device that is able to apply chemo-mechanical cues to keratinocytes and assess their proinflammatory cytokine IL-6 production. We showed that when chemical stimuli were applied with mechanical stimuli simultaneously, the IL-6 production markedly increased compared to that observed with a single stimulus. Quantitative structural analysis of cellular components revealed that the applied mechanical stretch transformed the cell morphology into an elongated shape, increased the cell size, and dictated the distribution of focal adhesion complex. Our results suggest that the mechanical cue-mediated modulation of focal adhesion proteins and actin cytoskeleton translates into intracellular signaling associated with the IL-6 production particularly in skin sensitization. Our study can be applied to understand proinflammatory responses of skin under altered biophysical environments of the skin.

## Introduction

Skin is exposed to various external biological or environmental factors (UV, nitric oxide or chemical energy)^1,2^, and a physical, chemical and immunological barrier to protect the body is the primary function of the skin. When workers or consumers are exposed to toxic chemicals in an accident or harmful cosmetics which cause allergies, the skin tissue is the first line of defense to be subject to chemical stimulants and it protects the body by activating inflammatory responses. In this situation, the skin tissue is exposed to mechanical stimuli as well as chemical stimuli simultaneously. The mechanical stimuli experienced by the skin tissue are more complex than other tissues in terms of magnitude and periodicity. The skin tissue may undergo various degrees of strain depending on the location^3^ or local deformation may occur due to skin diseases^4^. At cellular level, the internal and external mechanical forces acting on the skin tissue regulate cell proliferation, embryogenesis^5^, and wound healing^6^ through mechanotransduction-mediated signaling.

Keratinocytes as principal epidermal cells have been reported to produce many pro-and anti-inflammatory cytokines^7–9^. The proinflammatory cytokines by keratinocytes are upregulated by stimuli such as sensitizer^10–12^ and trigger the sensitization cascade. Therefore, many *in vitro* assays using dermal cells have been proposed to assess the inflammatory responses of skin cells to chemical stimuli^13,14^. The keratinocytes act as primary signal transducers releasing cytokines by chemicals^15^. Keratinocytes were cultured in a culture dish and their responses were observed after treating the cells with chemical stimulants, such as 2,4-dinitrochlorobenzene (DNCB) and hexyl cinnamic aldehyde^16,17^. However, these studies have limitations in investigating the effects of the dynamic environment of the skin involving stretching and bending of the skin, which are known to play an important role in the function of cells, on the inflammatory responses. In the aspect of mechanotransduction, it has been already reported that mechanical stretch plays a role in regulating the release of biomolecules including proinflammatory cytokines in types of cells. However, the effect of mechanical stimulation on dermal cells subject to chemical stimulant, which is a mechanically and chemically coupled stimulation, is still elusive.

We herein investigated the proinflammatory cytokine responses of human keratinocytes (HaCaTs) by treating the cells with chemicals and mechanical stretch, thus mimicking the physiological environment of the skin tissue. We investigated the effects of DNCB and mechanical stretch on the production of proinflammatory cytokine IL-6 by designing a uniaxial cell stretching system. In the 0–10% stretch region, IL-6 production was linearly proportional to the magnitude of the stretch in the absence of DNCB; however, when DNCB and stretch were applied simultaneously, the secretion of IL-6 rapidly increased, implying a synergistic effect of stretch and DNCB. IL-6 secretion was reduced when the magnitude of mechanical stretch exceeded 20%. In addition, we analyzed the morphological structure of keratinocytes by fluorescence imaging to identify if there was a correlation between immune response and changes of cell structure induced by stretch. Under proper intensity of stretch (0–10%), the cell area increased, and the cytoskeleton rearrangement was observed. At the excessive stretch (≥20%), the nucleus was increased and elogated, and vinculin was localized. The new experimental platform used in this study provides a comprehensive understanding of the inflammation responses of the skin tissue by observing how chemical and mechanical stimulation affect the inflammatory responses of human keratinocytes.

## Results

### Secretion of proinflammatory cytokine IL-6 by HaCaTs in response to DNCB

**Figure 1:**
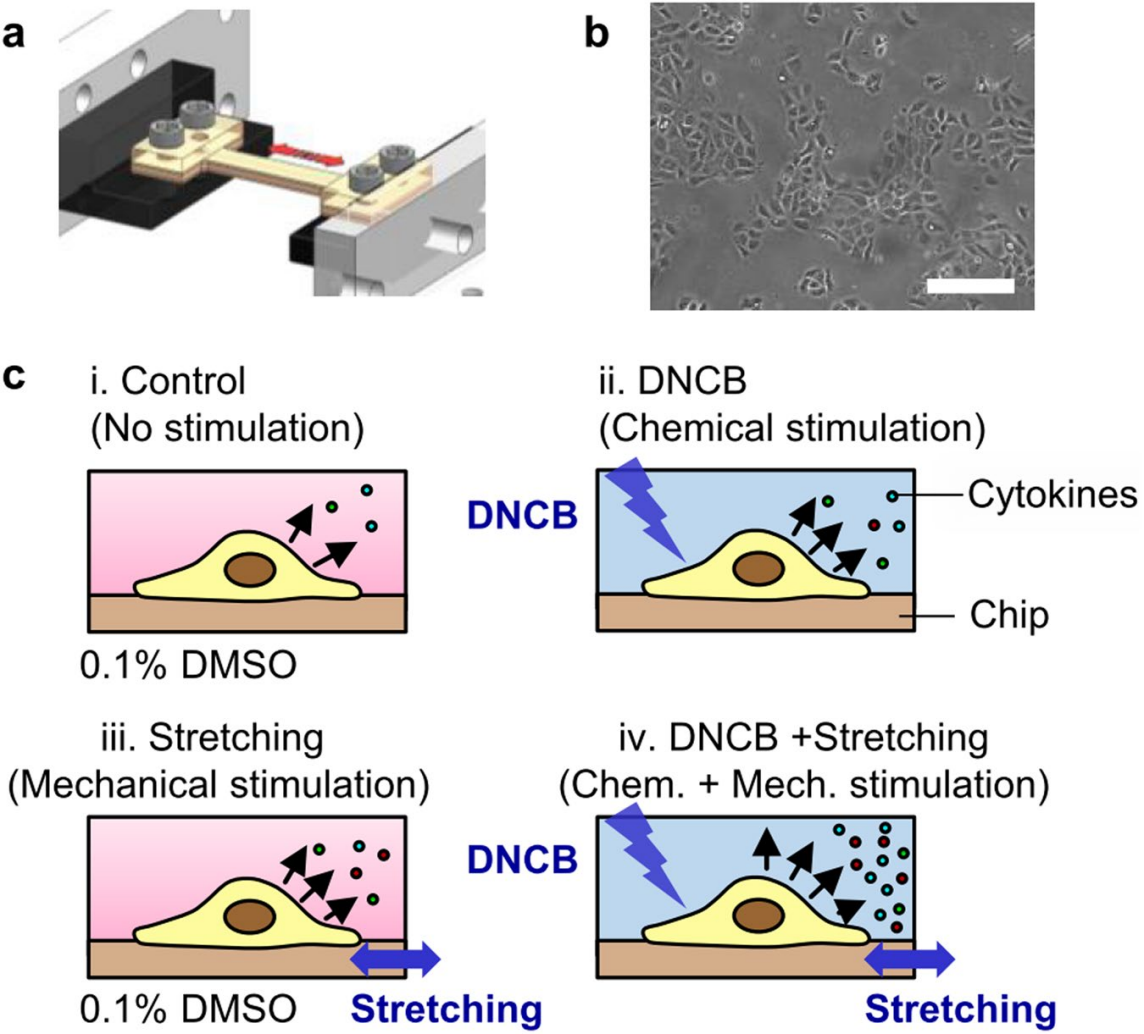
The experimental design for chemical and mechanical stimulation of proinflammatory response. (**a**) A schematic of the stretchable chip mounted on the custom-built uniaxial stretch chip (See Methods and Supplementary Fig. S1), (**b**) Image of HaCaT cells cultured on the stretchable chip after 24 h of preincubation. Scale bar represents 100 µm (**c**) An overview of the experimental design of the present work. All of the four conditions were conducted simultaneously in one experiment.

This study investigated the effects of each or both chemical stimulants and mechanical stretch on the production of proinflammatory cytokines in HaCaTs. The stretchable chip for cell culture was designed to control the chemical and mechanical environments of cells *in vitro* (Figs 1a and S1a). As shown in Fig. 1b, HaCaTs were seeded in the stretchable chip and the cells were incubated with a chemical stimulant and/or stretched cyclically. The I-shaped stretchable chip having a long channel in the stretch direction produced a uniform strain field in the entire channel, allowing the cells cultured in the chip to undergo the same magnitude of stretch. After stimulation, the supernatant was collected from the chip to quantify the level of proinflammatory cytokines produced by cells. To investigate the effect of chemical and mechanical stimulations on the proinflammatory response of HaCaTs, four experiment conditions were designed: (i) No stimulation (0.1% DMSO), (ii) Chemical stimulation (DNCB treatment), (iii) Mechanical stimulation (0.1% DMSO + cyclic stretch), and (iv) Chemo-mechanical stimulation (DNCB + cyclic stretch) (Figs 1c and S1b).

**Figure 2:**
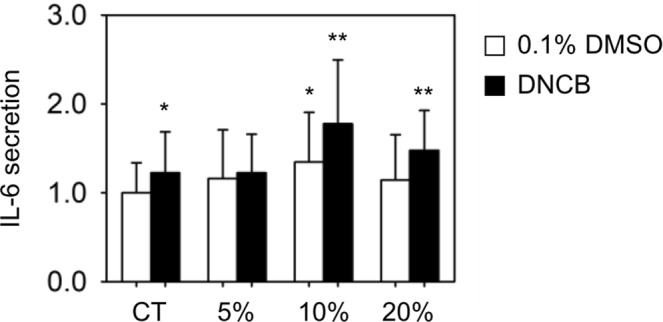
Effects of DNCB (5 μg/mL) and mechanical stretch on the production of proinflammatory cytokines in HaCaTs. Cells were cultured in the stretchable chips and were stimulated by chemical stimulants and/or mechanical stretch. The production of proinflammatory cytokines was quantified by ELISA, and all experimental values were normalized to the control group (CT) of each experiment. n = 35 for CT and DNCB, n = 9 for 5%, n = 9 for 5% with DNCB, n = 18 for 10%, n = 19 for 10% with DNCB, n = 12 for 20%, and n = 12 for 20% with DNCB. Values represent the mean ± SD. P values were calculated using t-test. * and ** mean P < 0.05 and P < 0.005, respectively.

To implement chemical stimulation, we used DNCB which has been widely used as a skin sensitizer. DNCB induces skin contact dermatitis^18^, and keratinocytes secrete proinflammatory cytokines in response to DNCB to promote inflammation^19^. In addition, we measured the proinflammatory cytokine responses induced by lactic acid (LA), a known non-sensitizer. Cells were cultured for 24 h in the stretchable chip before the induction of chemical stimulation (we called this as preincubation). After 24 h of preincubation, the culture medium was replaced with fresh cell culture medium supplemented with 0.1% DMSO (in the control group) or 5 μg/mL DNCB or 2000 μg/mL LA (in the chemical stimulation group). Cells were exposed to chemical stimulation for another 24 h, and the supernatant was collected to measure the level of proinflammatory cytokines by ELISA. To compare the inflammatory responses between experiment conditions easily, the production level of the proinflammatory cytokines was normalized by the average value of cytokines produced by the control group without any stimulation in each experiment. As results of chemical stimulation, IL-6 production by cells subjected to DNCB treatment increased 1.2-fold (Fig. 2); no DNCB dose-dependent IL-6 production was observed at the concentration range we used (Supplementary Fig. S2). However, no change in cytokine production was observed in non-sensitizer lactic acid treatment group (Supplementary Fig. S3). It is suggested that DNCB triggered the inflammatory response of HaCaTs through the production of the proinflammatory cytokine IL-6, which is induced by the activation of the immune system. It is consistent with previous reports that among the major cytokines produced by keratinocytes, DNCB treatment specifically increases the production of proinflammatory cytokines^20^. However, the increase level of IL-6 by DNCB was not as significant compared to the result observed in Jung *et al*.^20^, which might be due to the cell culture condition using a PDMS channel.

Regarding other representative proinflammatory cytokine IL-1α, HaCaTs produced IL-1α in response to chemical stimulants. However, the production of IL-1α by HaCaTs was low and slightly changed compared to IL-6 (Supplementary Fig. S3b), and thus, IL-6 was used as a biomarker to evaluate the proinflammatory response in this study.

### Effect of cyclic stretch on IL-6 production depending on the magnitude of stretch

Previous studies have shown that many cells exhibited an increased secretion of cytokines under the condition of mechanical stretch^21,22^. To evaluate the effect of mechanical stretch on the production of proinflammatory cytokines by HaCaTs, a cyclic stretch ranging from 5% to 20% in magnitude was applied at a fixed frequency of 0.5 Hz. Mechanical stimuli were set to replicate the physiological range of strain in the skin tissue^3^. After 24 h preincubation of cells in the stretchable chip, we replaced the supernatant with fresh cell culture medium supplemented with 0.1% DMSO to exclude DNCB-induced proinflammatory cytokine secretion. The cells were exposed to the culture medium for 24 h, and mechanical stretch was applied for the last 6 h. Since the cells respond to mechanical force within a few hours^23^, cells were mechanically stretched for a time shorter than chemical incubation time. Although chemical agents are specifically attached to cell membrane ligands, mechanical forces can induce signaling in a non-specific manner through relatively diverse signaling pathways.

As shown in Fig. 2, HaCaTs produced IL-6 in dependent with the magnitude of stretch. Higher level of IL-6 production was shown in mechanical stretch-induced cells compared to those without stretch. In the presence of 5% and 10% of stretch, IL-6 production increased by 1.2-fold and 1.3-fold, respectively, which is consistent with previous studies showing that the secretion of proinflammatory cytokines increases as the magnitude of mechanical stimulation increases^24^. However, the production of IL-6 level increased by only 1.1-fold at 20% stretch compared to that without stretch. When <10% stretch was applied to the cells, the production of IL-6 increased with increasing the magnitude of stretch; however, the production of IL-6 decreased at ≥20% stretch.

### The dramatic increase in IL-6 production by the coupled effect of cyclic stretch and DNCB

To determine the coupled effect of mechanical stretch on DNCB-induced IL-6 production, cells cultured in the stretchable chip were simultaneously exposed to both chemical stimulant and mechanical stretch. As in the previous experiments, HaCaTs were preincubated for 24 h in stretchable chips, and then, we replaced cell medium with the culture medium supplemented with DNCB. Cyclic stretch was applied during the last 6 h of the 24 h treatment of DNCB, and the supernatant was collected to measure the level of cytokines accumulated during 24 h of chemical and mechanical stimulation.

Simultaneous stimulation using mechanical stretch and DNCB exhibited the different IL-6 response compared to those seen when only either DNCB or mechanical stretch was applied. As shown in Fig. 2, when the cells were subjected to both 5% mechanical stretch and DNCB treatment simultaneously, the production of IL-6 increased by 1.2-fold higher than unstretched. It is the similar amount of IL-6 produced under 5% stretch or DNCB treatment only. It suggested that 5% mechanical stretch and DNCB increase the secretion of IL-6, respectively; however, when both stimulations are applied to cells at the same time, two stimulations may balance, not leading to a further increase of IL-6. In contrast, when 10% mechanical stretch was applied to DNCB-induced cells, IL-6 secreation increased by 1.8 folds indicative of a synergistic effect. For 20% stretch, IL-6 was increased significantly (1.5-fold increase) when both chemical and mechanical stimuli were applied simultaneously compared to the increase by the 20% mechanical strech itself (1.1-fold increase). Therefore, the simultaneous stimuatlion of mechanical stretch and DNCB produces a synergistic effect in the inflammatory response of the skin tissue at 10% and 20% strain, and the IL-6 production vary depending on the magnitude of stretch.

### Morphological changes of cell and nucleus regulated by cyclic stretch and DNCB

**Figure 3:**
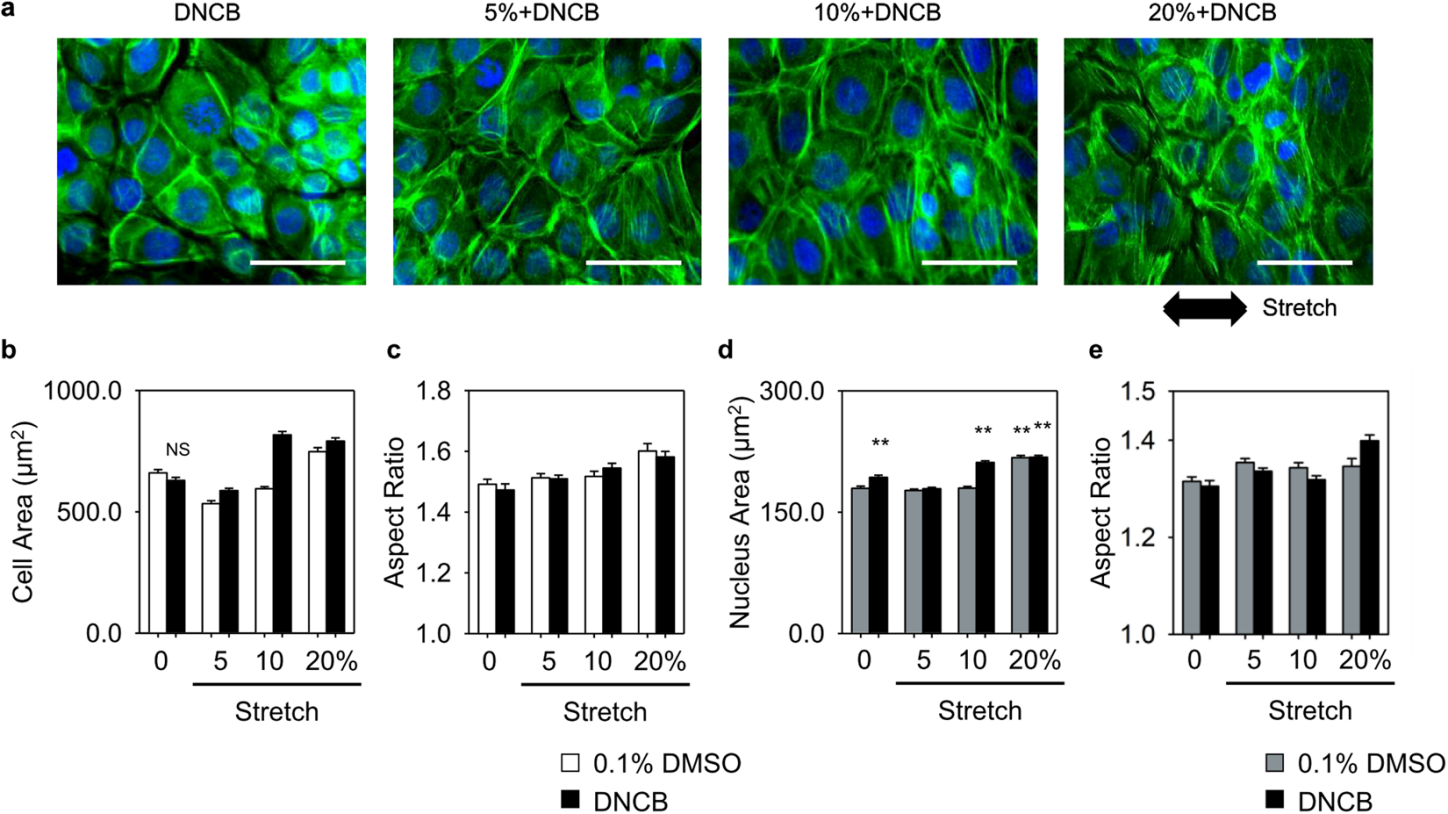
Mechanical stretch induces cytoskeletal remodeling which changes cell morphology. (**a**) Representative fluorescent images showing the nucleus (blue) and actin (green) under DNCB treatment and mechanical stretch with varying strain. Scale bars represent 50 µm. Mechanical stretch regulated the area (**b**) and aspect ratio (**c**) of cell. The area (**d**) and aspect ratio (**e**) of nucleus depending on the magnitude of stretch. All images were analyzed using the Cell Profiler software according to the sequence shown in Supplementary Fig. S4. Error bars for (**b**–**e**) indicate SEM. n = 350–1000 in (**b**–**e**). P values were calculated using t-test. ** and NS means P < 0.005 and not significant (P > 0.05), respectively.

The mechanical forces applied to cells are transduced into biological signals through integrin-mediated mechanotransduction^25^. Alteration of integrin causes morphological changes in cells, and focal adhesion (FA) complex assembly acts as a mechanosensitive sensor in stretch-induced mechanotransduction^26^. To investigate the effects of cyclic stretch and DNCB treatment on the cell structure, area and shape of cell and nucleus, and size and orientation of focal adhesion were characterized. We used fluorescent images to visualize the changes in HaCaT cytoskeletal structure in response to cyclic stretch with DNCB treatment. The images were quantitatively analyzed (Figs 3a and S4) using Cell Profiler^27^, and the alterations in the area and orientation of cell and nucleus were estimated as a function of stretch magnitude.

The area of cells treated with DNCB was the same as that of unstretched cells (Fig. 3b, 660 ± 13 μm^2^ for the control group, 629 ± 12 μm^2^ for DNCB, mean ± SEM values); however, the area of nucleus was increased by 8% (180 ± 3 μm^2^ for the control group, 193 ± 3 μm^2^ for DNCB, mean ± SEM values). When only mechanical stretch was applied, the nucleus area did not increase at stretch < 20% and it increased significantly at 20% stretch (Fig. 3d). Under simultaneous stimulation of DNCB and mechanical stretch, there was an increase in cell area compared to the experimental results for only mechanical stimulation with the same magnitude of stretch. In particular, at 10% stretch with DNCB treatment, the areas of both cell and nucleus were greatly increased compared to the results obtained when only mechanical stretch was applied (cell: 595 ± 9 μm^2^ for 10%, 817 ± 14 μm^2^ for 10% + DNCB; nucleus: 180 ± 2 μm^2^ for 10%, 211 ± 2 μm^2^ for 10% + DNCB; mean ± SEM values). And as the magnitude of the mechanical stretch increased, the cell and nucleus were elongated (Fig. 3c,e). It was observed that the area of cells was decreased at 5% stretch; however, the cells expanded as the magnitude of stretch increased. The shrinkage of cells observed at 5% is in agreement with previous reports where the area of cells decreased under mild mechanical stretch but increased with continuing duration of stretch^28^. Thus, it can be assumed that when mild stretch is applied to the cells, they undergo a transient process wherein they are rearranged and subjected to various changes.

HaCaTs showed reorientation of both cell and nucleus on exposure to cyclic stretch (Supplementary Fig. S5). Both cell and nucleus were oriented perpendicular to the direction of stretching. The orientation of unstretched cells with DNCB treatment was evenly distributed from 0° to 180°; however, the orientation of stretched cells was observed to be distributed in the 90° region, and the cells were more aligned at 90° (perpendicular to the stretching direction) as the magnitude of stretch increased.

**Figure 4:**
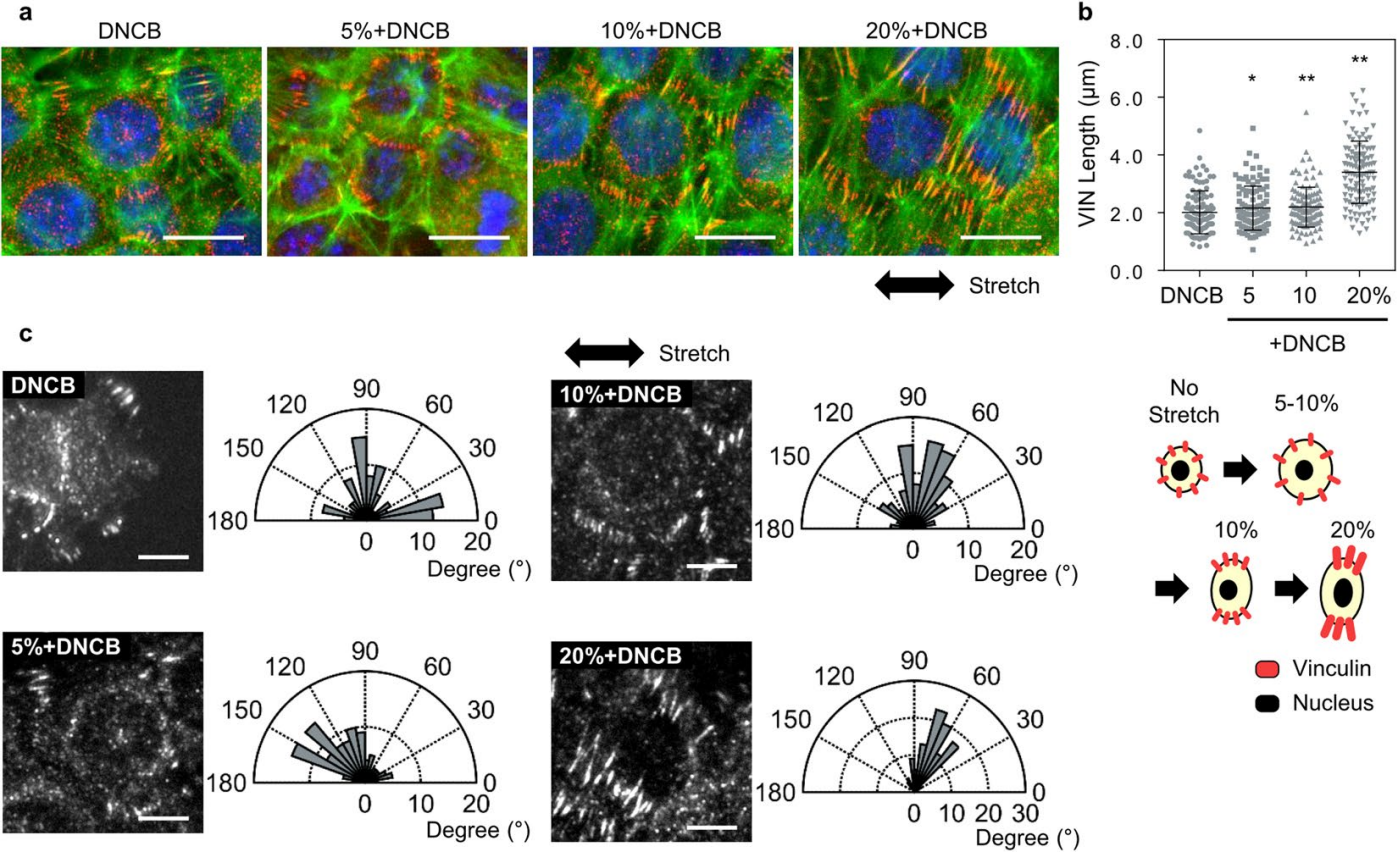
Redistribution of vinculin (VIN) in HaCaTs in response to chemical and mechanical stimulation. Mechanical stretch applied to cells transduced via focal adhesion in the sites of integrin which initiates downstream signaling. (**a**) Representative images for vinculin in response to DNCB and mechanical stretch. Scale bars are 20 μm. (**b**) VIN length as a function of stretch magnitude. P values were calculated using t-test. * and ** mean P < 0.05 and P < 0.005, respectively. (**c**) Immunostained images of vinculin and its orientation. Focal adhesion was reoriented along 90° (perpendicual to the stretching direction). Scale bars represent 10 µm. n = 105–119 in (**b** and **c**).

Next, we observed a change in the focal adhesion (FA) complex of HaCaTs in response to mechanical stretch (Fig. 4a). When mechanical stimulation is applied, the forces are transmitted into biochemical signals via dynamic protein complex of focal adhesion that regulates cell attachment to the substrate. Mechanical stretch has been previously reported to increase the length of FA and to regulate the cell orientations^29,30^. In this study, vinculin was used as a focal adhesion marker and was immunostained to study the effect of mechanical stretch on FA dynamics. The lengths and orientations of vinculin were measured by manually determining the appropriate threshold intensity of images in ImageJ software (Supplementary Fig. S6). Quantitative analysis of images revealed the localization of vinculin to FAs in cells and a drastic increase in FA lengths with increasing magnitudes of stretch. At 5% and 10% stretch, the length of vinculin was 2.16 μm and 2.19 μm, respectively; however, a significant increase by 1.65-fold (3.40 μm) at 20% stretch was observed (Fig. 4b). Vinculin was also oriented perpendicular to the direction of stretch, and the distribution was similar to that observed in cells treated with an increased magnitude of stretch (Fig. 4c). It was not observed that cells were dead by 20% excessive mechanical stimulation (Supplementary Fig. S7).

Taken together, under DNCB treatment and mechanical stretch, as the magnitude of the mechanical stretch increased, the cells became larger and more elongated and showed perpendicular alignment to the stretching direction. In particular, under 20% stretch and DNCB treatment, the area and the major axis length of the nucleus were increased and vinculin showed a high localization.

Changes in cell area were similar to those in IL-6 production. As the magnitude of the stretch increased, IL-6 production by cells gradually increased, and at simultaneous stimulation of DNCB and 10% stretch, both cell area and IL-6 production increased significantly. An increase of the nucleus area was also observed at 10% stretch with DNCB treatment. However, IL-6 production decreased at 20% stretch, whereas the cell area increased. It has been suggested that an excessive mechanical stretch may have a different effect on the production of cytokines as observed in the increase of the nucleus area.

## Discussion

Treatment of cells with toxic chemicals activates the inflammatory responses. Also, many studies have reported that mechanical stimulation applied to cells regulates the production of inflammatory cytokines in cells. Despite the fact that both stimuli can affect the inflammatory response of cells and those are simultaneously present in the physiological environment of the tissue, the effects of chemical or mechanical stimuli have only been studied separately in previous studies. Therefore, it is important to study the effects of mechanical stimuli on chemical stimuli-induced inflammatory responses to study immune responses in the skin which is continuously exposed to mechanical stimuli. We applied sensitizing chemicals and cyclic stretch to cells to investigate the inflammatory response of cells to sensitizer in biophysical environments of the skin.

When mechanical stretch and DNCB simultaneously stimulated the cells, IL-6 secretion increased more compared to only mechanical stretch or DNCB treatment. Unlike the results of chemical or mechanical individual stimulation, simultaneous stimulation was shown to lead to synergistic effects in the cytokine production. This result may represent the inflammatory response in the physiological environment of the skin tissue better. We suggest that different inflammatory responses may occur depending on the magnitude of the stretch in relation to the pathological condition of the skin tissue. The role of mechanical force in the process of wound healing and formation of scars is important^6,31,32^, and the strain-dependent inflammatory responses can affect those processes. Applying stretch to a tissue with an altered structure by a wound, cells undergo different magnitudes of stretch depending on their location and they can exhibit different inflammatory responses. The stiffness of hypertrophic scar or keloid caused by angiogenesis are higher than that of the normal skin tissue. When the skin is stretched, the magnitude of stretch is low on the scar but is high at the boundary. This scar–strain relation suggests that the scar and the skin around the scar can show different inflammatory responses.

The forces exerted outside the cell are transferred to the inside of the cell through the connection of the integrin–focal adhesion complex-bundled actin filament (or stress fiber), which changes the structure of the cell. The actin cytoskeleton is reportedly associated with the secretion of cytokines. The depolymerized actin cytoskeleton network of human endothelial cells using cytochalasin D reduces the production of IL-8 and MCAF/MCP-1^33^. The treatment of chondrocytes with a chemical agent, such as IL-1, increases the amount of fibrous actin and regulates the cytoskeletal tissue^34^. These indicate that the actin cytoskeleton, which plays an important role in regulating cell morphology, is closely related to the inflammatory response. We also found that as the magnitude of the stretch increased, the shape became more elongated and the orientation rearranged. This finding is consistent with the results of previous studies that under cyclic stretching, cells are rearranged to minimize the effects of external deformation^35,36^. In our study, the area of the cells was not increased when only chemical stimulus was applied; however, it was slightly increased when both mechanical stretch and DNCB treatment were applied together. A drastic increase in the nuclei area was observed under 10% stretch with DNCB treatment. We found that the trend of increasing and decreasing cell area was similar to that of the change in IL-6 production in response to various magnitudes of stretch. Based on the hypothesis that there is a relationship between the cell area and IL-6 production, we proposed that the signaling pathways triggered by DNCB and by mechanical stretch act synergistically. One possibility is that as the cell area increases, the DNCB binding probability increases, and at the same time, mechanical stretch can activate signal pathways that are sensitive to integrin-based mechanical signals. Conversely, when excessive stretch of ≥20% strain was applied to the cells, the area of cell and nucleus increased immensely, and focal adhesion formed by vinculin was highly localized. At 20% stretch, the IL-6 production reduced, indicating that the mechanical forces on a cell were excessive. Excessive mechanical forces transmitted through the actin cytoskeleton can alter the shape of the nucleus, which can affect gene expression. Cytoskeletal prestress has been reported to alter the shape of the nucleus^37^, and gene expression can be altered if the nucleus structure is modified in response to external stimuli^38^. Thus, the cytoskeleton can play an important role in activating various signaling pathways that can affect the inflammatory response of the cells by modulating gene expression through the nucleus deformation.

**Figure 5:**
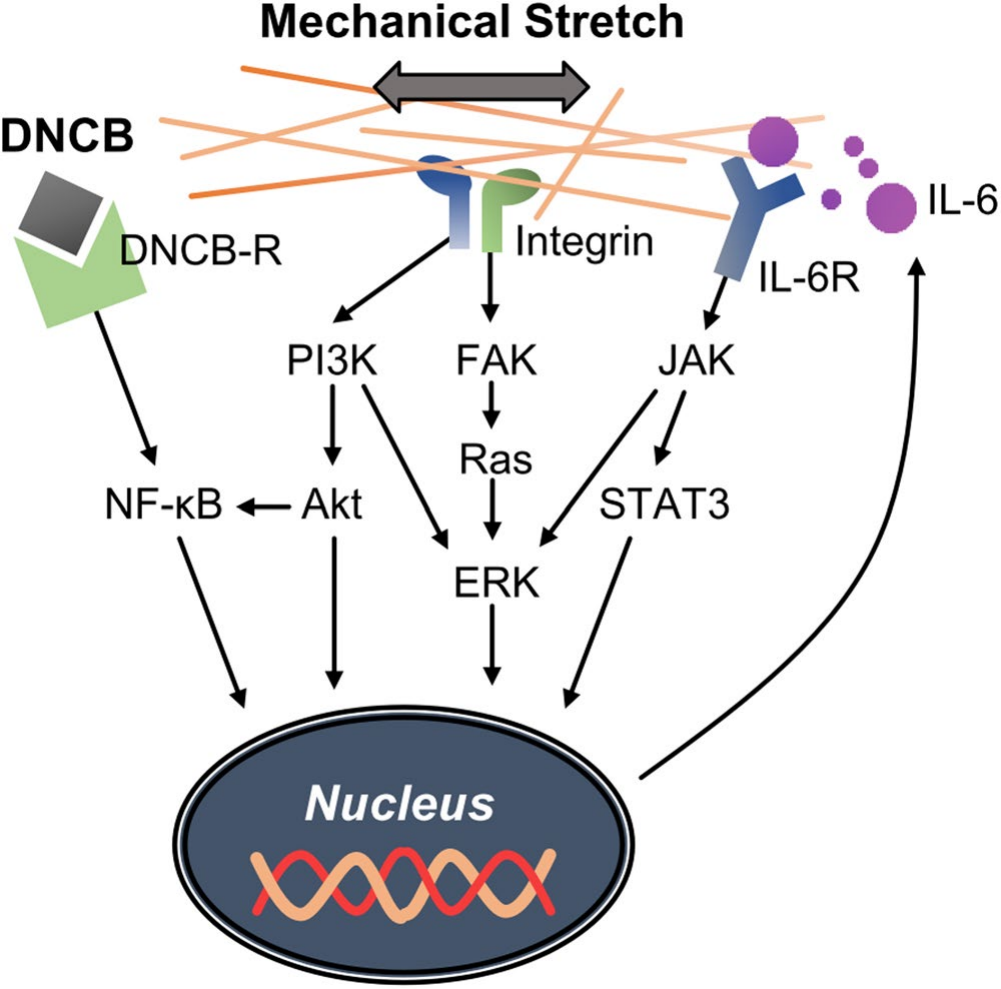
The mechano-chemical transduction signaling pathways participating in the production of IL-6. A combinatorial pathway in response to mechanical and chemical stimulation is suggested to explain their synergetic effect in IL-6 secretion.

The biophysical cues, particularly the mechanical stretching applied to the cells, are transmitted to the cells via the integrin–actin cytoskeleton; these cues alter gene expression owing to the deformation of the nucleus activating various signaling pathways. Figure 5 shows the possible pathway that is mainly involved in mechanotransduction and chemical sensitizing^39–49^. The signaling pathways described in Fig. 5 are some of the candidates that explain the synergistic effects of mechanical stretching and chemical stimuli observed in IL-6 production in keratinocytes. Inhibition tests of potential mechanistic pathways showed how the mechanosignaling and DNCB signaling pathways are interrelated (Supplementary Fig. S8). NF-κB is directly involved in chemical stimulation and the mechanical stretch can be transmitted through the PI3K signaling pathway. ERK or STAT3 appear to be involved in the synergistic effect of simultaneous chemical and mechanical stimulation.

The synergistic effect of chemical and mechanical stimulation on IL-6 secretion were also observed in *ex vivo* experiments. Under chemical and mechanical simultaneous stimulation, IL-6 secretion was significantly increased compared to mechanical or chemical sole stimulation (Supplementary Fig. S9). Since the signaling pathways involved in the immune responses are complex, it is difficult to elucidate the exact mechanism underlying the chemo-mechanical synergistic effect. However, the synergistic effect of mechanical-chemical stimulation investigated in this study suggets that mimicking the mechanical environment of the skin is necessary to study the inflammatory response.

## Methods

### Cell culture

The human keratinocyte cell line HaCaT was provided by the German Cancer Research Center (Heidelberg). HaCaT cells were cultured in Dulbecco’s modified Eagle medium, which was supplemented with 10% heat-inactivated fetal bovine serum (Invitrogen), 1% penicillin–streptomycin (Invitrogen), and 1% non-essential amino acids (Gibco). Cells were cultured at 37 °C in a humidified incubator containing 5% CO_2_.

### Chemical stimulant

The skin cells cultured on stretchable chips were treated with 5 μg/mL 2,4-dinitrochlorobenzene (DNCB, Sigma-Aldrich) as a sensitizer. DNCB was dissolved in dimethyl sulfoxide (the maximum concentration of DMSO in the culture medium was 0.1%), and then diluted in cell culture medium. The culture medium supplemented with 0.1% DMSO was used as a vehicle control. The concentrations of DNCB and lactic acid used in this study were determined based on prior tests of 75% cell viability (CV_75_), as described previously^20^.

### Cyclic stretch experiment

With the stretchable chip, cells were exposed to chemical stimuli and mechanical stretch either individually or simultaneously. To apply mechanical stimulation to cells, the stretchable chip was fabricated usingpolydimethylsiloxane (PDMS) with a mixing ratio of 10:1 (polymer: curing agent, w/w). The stretchable chip comprised four layers: blocks, upper, middle, and lower layers. The thicknesses of the upper and lower layers and that of the middle layer were 500 μm and 1 mm, respectively. Layers were cut by custom-designed cutter, and the size of cell culture channel was 3 mm (W) × 33 mm (L) × 1 mm (H). All layers were attached to each other by oxygen plasma treatment (Femto Science) and manually aligned using a microscope. Blocks of 5 mm thickness were attached to both ends of the stretchable chip to prevent the deformation at the screwed region when the chip was fixed to the linear stage. Cells were seeded in the channels through the inlet and outlet at both ends of the chip, and the culture media, chemicals, and supernatants were introduced and collected through the inlet and outlet, respectively. The surface of the channel was functionalized overnight with 2.5 μg/mL fibronectin at 4 °C for cell culturing.

The stretch device was equipped with a linear stage (Oriental Motor Inc.) and a customized fixture that was capable of applying uniaxial stretching to multiple cell chips simultaneously (Fig. S1). The cyclic stretch was applied for 6 h at 0.5 Hz. The amplitude of strain was varying in the range of 5–20% similar to the strain underwent by cells *in vivo*.

### Cytokine production by ELISA

The incubation time of the chemical stimulant applied to the cells was as described previsouly^20^. After 24 h of incubation under chemical or mechanical stimulation, the cell culture media were analyzed using ELISA for IL-6 and IL-1α (R&D Systems), according to the manufacturer’s instructions.

### Immunofluorescence cell staining and image analysis

Cells were fixed in 4% paraformaldehyde (Electron Microscopy Science) and permeabilized in 0.5% Triton X-100 (Sigma-Aldrich). For actin and nucleus staining, cells were incubated with Alexa-488-labeled phalloidin (Invitrogen) and Hoechst 33342 (Sigma-Aldrich) in 1% BSA (Sigma-Aldrich) blocking solution. For focal adhesion staining, cells were incubated with rabbit anti-vinculin (Invitrogen) primary antibody for 1 h at room temperature. After washing, cells were incubated for 1 h with Alexa-568-labeled goat anti-rabbit secondary antibody. All images were obtained using an upright microscope (Nikon) with Plan Fluor 20 × (NA 0.5) and S Plan Fluor 40 × (NA 0.6) objectives.

### *Ex vivo* experiments

Tissue flap containing dermis, subcutaneous muscle, and subcutaneous tissue was excised from the back of C57BL/6 mice and a 2.5 cm × 2 cm tissue was loaded on the stretch device. Tissue was stretched at 20% strain for 3 h and 250 mg/mL of DNCB was treated during stretching. After chemical and mechanical stimulation, the tissues were cut and RT-PCR was performed. All process from tissue incision to tissue loading on the stretch device was performed within 3 h. Detailed experimental methods are described in Supplementary Information.

*Ex vivo* experiments were conducted in accordance to the Institute for Experimental Animals College of Medicine and the experiment protocols were approved by the Institutional Animal Care and Use Committee of Seoul National University (accession number SNU-181030-1).

### Statistical analysis

All statistical analyses were run using GraphPad Prism 5.0 and Sigma Plot 10.0 software. A statistical significance of the difference between data was assessed by calculating the P value using two-tailed Student t-test or ANOVA.

## Supplementary information

Supplementary information
