# Supplementary material for: Effect of Mechanical Stretch on the DNCB-induced Proinflammatory Cytokine Secretion in Human Keratinocytes
Source: Sci Rep. 2019 Mar 26;9:5156. doi: 10.1038/s41598-019-41480-y (PMC6435715; doi:10.1038/s41598-019-41480-y)
Supplement: Supplementary file 1 — Supplementary information [file 41598_2019_41480_MOESM1_ESM.docx]

**Supplementary Information**

**Effect of Mechanical Stretch on the DNCB-induced Proinflammatory Cytokine Secretion in Human Keratinocytes**

Seunghee Oh^1,3^, Hyewon Chung^2^, Sooho Chang^1^, Su-Hyon Lee^4^, Seung Hyeok Seok^2^, and Hyungsuk Lee^1^

^1^School of Mechanical Engineering, Yonsei University, Seoul, 03722, South Korea

^2^Department of Microbiology and Immunology, and Institute of Endemic Disease, College of Medicine, Seoul National University, Seoul, 03080, South Korea

^3^Global Technology Center, Samsung Electronics, Co., Ltd., Suwon, 16677, South Korea

^4^R&D Institute, Biosolution Co., Ltd., Seoul 01811, South Korea

Correspondence and requests for materials should be addressed to S.H.S. (email: lamseok@snu.ac.kr) or H.L. (email: hyungsuk@yonsei.ac.kr).


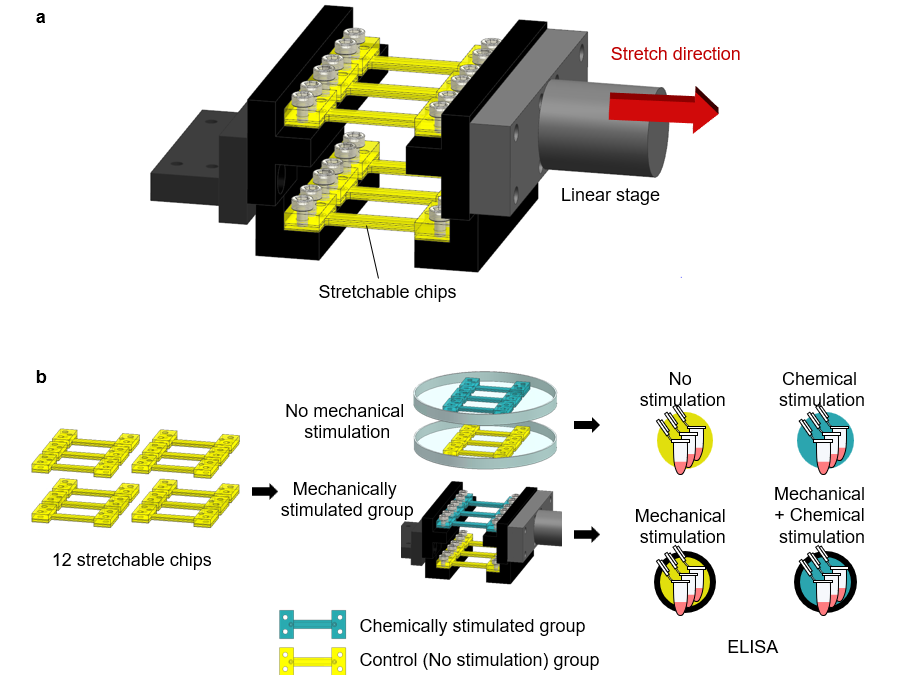


**Figure S1. Uniaxial stretching chip and characterization.** (a) 3D schematic of the stretchable chips loaded on the stretching device. A linear stage was used for uniaxial stretch and the six stretchable chips were held by screws onto the fixture to perform mechanical stimulation by stretching. (b) Schematic of one experiment set. One experimental set was executed in the same day. The average value of cytokine production of the control group (no stimulation) was calculated and all cytokine production was normalized to this average value.


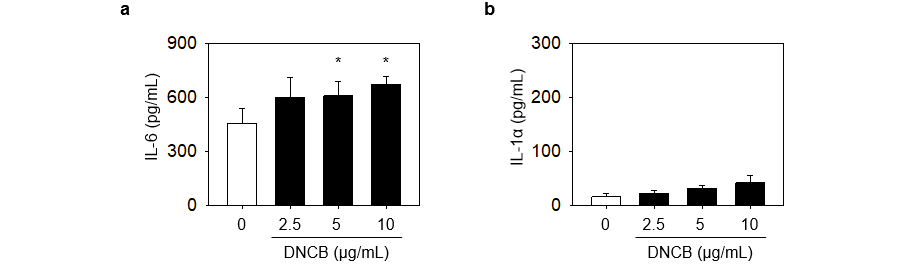


**Figure S2. DNCB-dose dependent cytokine production level**. (a) Production of IL-6 and (b) IL-1α in response to DNCB. Values represent the mean ± SD. P values were calculated using t-test, *, P < 0.05.


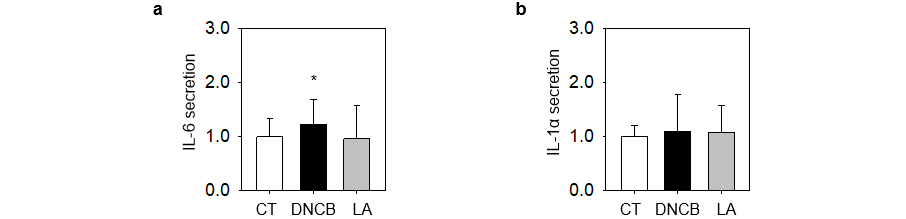


**Figure S3.** **Cytokine production level on chemical stimulation.** (a) Production of IL-6 in response to DNCB and lactic acid (b) Production of IL-1α in response to DNCB and lactic acid. Values represent the mean ± SD. P values were calculated using t-test, *, P < 0.05.


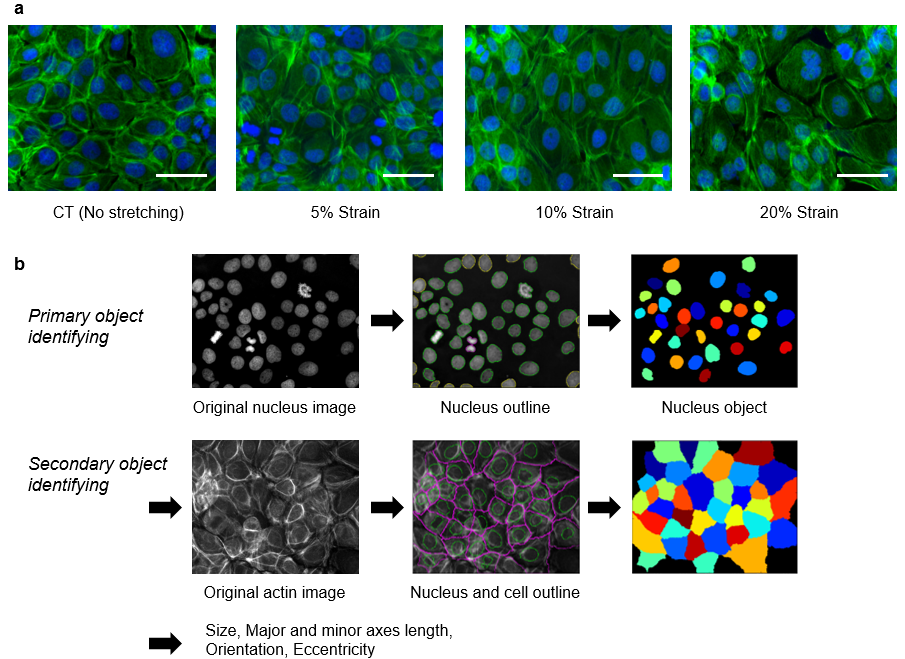


**Figure S4. Quantification of cytoskeletal structure using Cell Profiler pipeline.** (a) Representative fluorescent images of HaCaTs showing nuclear (blue) and actin (green) under mechanical stretch with varying the magnitude of stretch. Scale bars, 50 µm (b) A schematic for analyzing the process of cells. Fluorescent images were taken and size and morphology were analyzed using Cell Profiler software (www.cellprofiler.org).


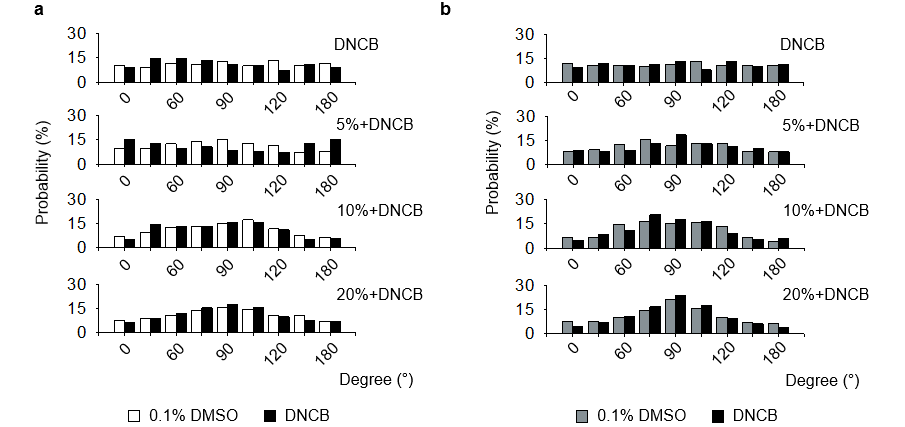


**Figure S5. Mechanical stretch changes cell and nucleus orientation morphology.** Cell orientation distribution (a) and nucleus orientation distribution (b) in response to chemical and mechanical stimulation. Cells and nucleus were reoriented perpendicular to the direction of stretch with increase in strain.


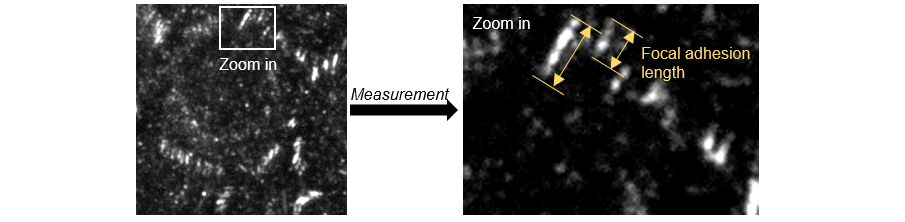


**Figure S6. Quantification of focal adhesion length using ImageJ.**


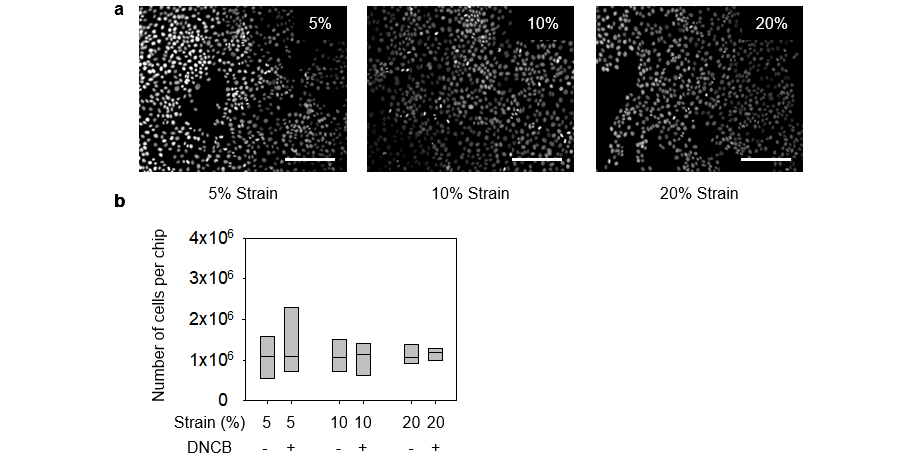


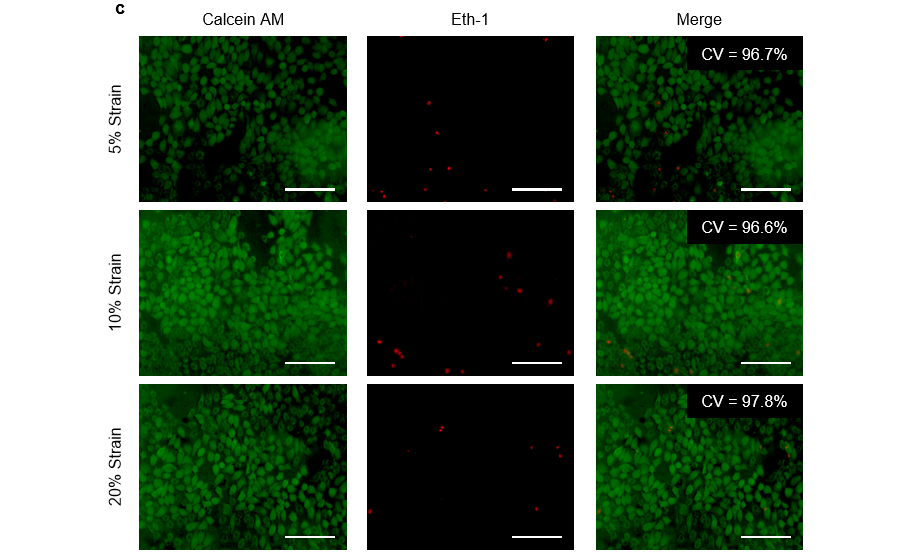


Figure S7. The number of cells cultured in the stretchable chips and cell viability. The number of cells cultured in the stretchable chip after the DNCB stimulation and mechanical stretching with different strain magnitudes (5%, 10% and 20%) was counted using stained nuclei. Images were captured at three or four different locations on a chip in more than three chips per condition. (a) Representative stained nucleus images for 5%, 10%, and 20% strain and (b) the number of cells in one stretchable chip after chemical and mechanical stimulation. (c) Live/dead staining using calcein AM and ethidium homodimer 1 after 5%, 10% and 20% stretching. Cells were labeled with calcein-AM and ethidium homodimer-1 (#L3224, LIVE/DEAD™ Viability/Cytotoxicity Kit, Molecular probes). Cell viability of 20%-stretched cell (97.8%) was similar to 5% and 10%-stretched cells (96.7% and 96.6% respectively), and suggesting that no cell death suspected by excessive stimulation was observed. Scale bars are 100 μm.


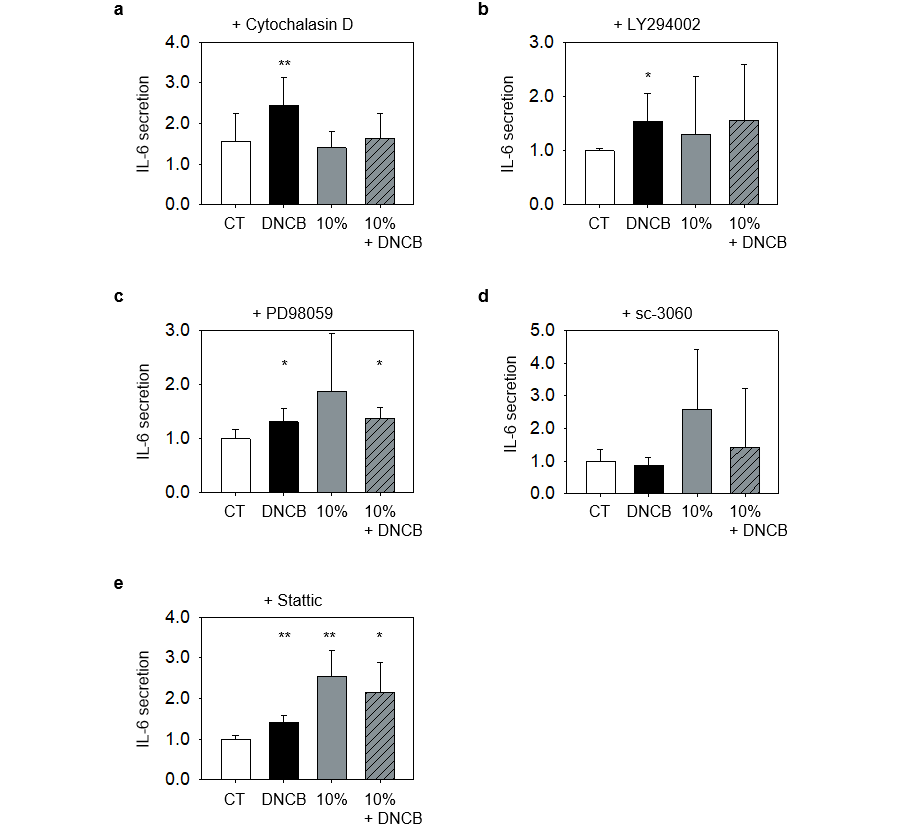


**Figure S8.** **Inhibition test results.** Actin polymerization (a), PI3K-Akt (b), ERK (c), NF-κB (d), and STAT3(e) in HaCaT cells were inhibited and 10% stretch and DNCB stimulation-induced IL-6 production levels were quantified. The inhibitors were used at the following concentrations: cytochalasin D, 20 μM; LY294002, 10 μM; PD98059, 50 μM; sc-3060, 100 ng/mL; Stattic, 5 μM. Values represent the mean ± SD. P values were calculated using t-test, *, P < 0.05, **, P < 0.005.


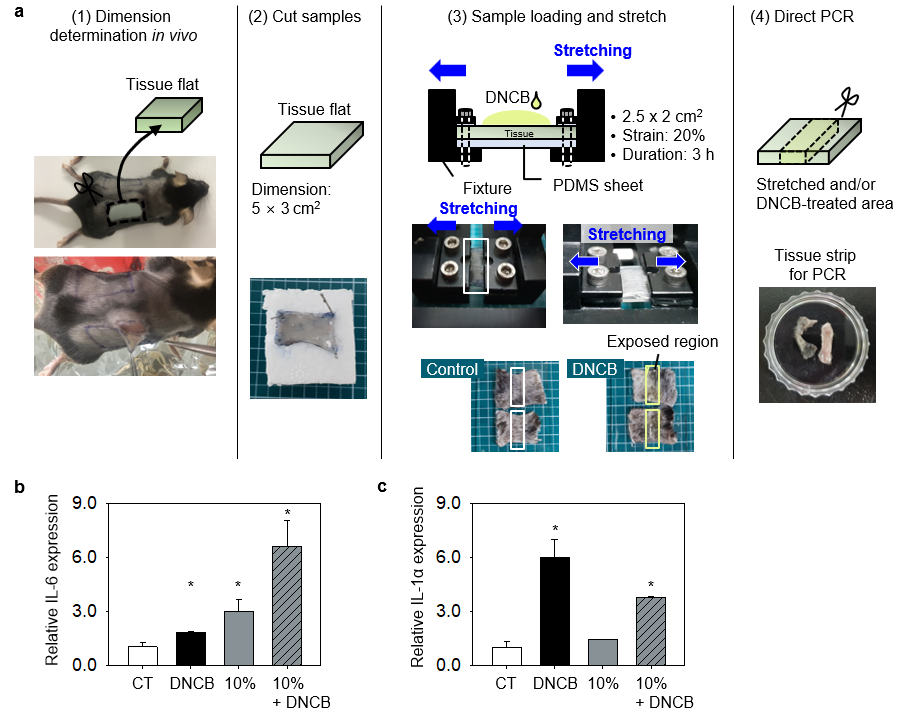


**Figure S9. Ex vivo experiment results.** (a) Experimental setup of skin tissue stretch *ex vivo*. (b) Quantitative RT-PCR results of IL-6, and (c) IL-1α expression. Values represent the mean ± SD. P values were calculated using t-test, *, P < 0.05.

**Methods**

**Inhibition test**

Actin polymerization inhibitor Cytochalasin D, PI3K-Akt inhibitor, LY294002, and ERK inhibitor, PD98059, NF-κB inhibitor sc-3060, and STAT3 inhibitor, Stattic were employed and measured IL-6 secretion of HaCaT by ELISA. After 23 h preincubation, cells were pretreated with inhibitors for 1 h before chemical and mechanical stimulation. All inhibitors were added to the medium. After pretreatment culture medium supplemented with inhibitor and 0.1% DMSO or DNCB was replaced.

***Ex vivo* experiments**

Immediately after death, an 5 cm × 3 cm tissue flap containing dermis, subcutaneous muscle, and subcutaneous tissue was excised from the back of C57BL/6 mice (19–24 g). The transverse length of the excised tissue sample was measured and marked on the skin of the mouse before excision. After dissection, the tissue flap’s transverse length was measured again and cut into a size of 2.5 cm × 2 cm. The tissue was loaded on the stretch device with screws fixed on both sides. Except for fixed area, a 4 mm wide strip area was exposed and 250 mg/mL of DNCB was treated to this area. For no-stretch group (control and DNCB treatment group), the film was covered both side of tissue to expose 4 mm-wide strip area in the tissue. Tissue was stretched in 20% strain for 3 h and DNCB was treated during stretching. Since the tissue is a complex structure that composed of multiple layers of cells and extracellular matrix and cells in tissue undergo different strain. Therefore, to see the effect of mechanical stretch on the inflammatory response of tissues, the tissue was stretched in 20% strain, which is larger strain than that was conducted in cell experiments. After all the experiments, the tissues of the 4 mm strip area where mechanical and chemical stimuli were applied were cut and RT-PCR was performed. All process from tissue incision to tissue loading on the stretch device was performed within 3 h.

**Real-time quantitative PCR**

RNA was extracted with TRIzol (Invitrogen) according to the manufacturer’s instruction. cDNA was synthesized from 1 µg of total RNA and level of mRNA was determined by quantitative real-time PCR using the SYPB Green PCR Master Mix (Applied Biosystems). Primer sequences were as follows: human IL-6 (forward: 5'-AAA TTC GGT ACA TCC TCG AC-3', reverse: 5'-CAG GAA CTG GAT CAG GAC TT-3'), human IL-1α (forward: 5'-AGT AGC AAC CAA CGG GAA GG-3', reverse: 5'-TGG TTG GTC TTC ATC TTG GG-3'), human actin (forward: 5'-TCT ACA ATG AGC TGC GTG TG-3', reverse : 5'-ATG GCT GGG GTG TTG AAG-3').
